# GEneSTATION 1.0: a synthetic resource of diverse evolutionary and functional genomic data for studying the evolution of pregnancy-associated tissues and phenotypes

**DOI:** 10.1093/nar/gkv1137

**Published:** 2015-11-14

**Authors:** Mara Kim, Brian A. Cooper, Rohit Venkat, Julie B. Phillips, Haley R. Eidem, Jibril Hirbo, Sashank Nutakki, Scott M. Williams, Louis J. Muglia, J. Anthony Capra, Kenneth Petren, Patrick Abbot, Antonis Rokas, Kriston L. McGary

**Affiliations:** 1 Department of Biological Sciences, Vanderbilt University, Nashville, TN 37235, USA; 2 Department of Genetics, Geisel School of Medicine, Dartmouth College, Hanover, NH 03755, USA; 3 Center for Prevention of Preterm Birth, Perinatal Institute, Cincinnati Children's Hospital Medical Center, Cincinnati, OH 45229, USA; 4 Department of Biomedical Informatics, Vanderbilt University Medical Center, Nashville, TN 37235, USA; 5 Department of Biological Sciences, University of Cincinnati, Cincinnati, OH 45221, USA

## Abstract

Mammalian gestation and pregnancy are fast evolving processes that involve the interaction of the fetal, maternal and paternal genomes. Version 1.0 of the GEneSTATION database (http://genestation.org) integrates diverse types of omics data across mammals to advance understanding of the genetic basis of gestation and pregnancy-associated phenotypes and to accelerate the translation of discoveries from model organisms to humans. GEneSTATION is built using tools from the Generic Model Organism Database project, including the biology-aware database CHADO, new tools for rapid data integration, and algorithms that streamline synthesis and user access. GEneSTATION contains curated life history information on pregnancy and reproduction from 23 high-quality mammalian genomes. For every human gene, GEneSTATION contains diverse evolutionary (e.g. gene age, population genetic and molecular evolutionary statistics), organismal (e.g. tissue-specific gene and protein expression, differential gene expression, disease phenotype), and molecular data types (e.g. Gene Ontology Annotation, protein interactions), as well as links to many general (e.g. Entrez, PubMed) and pregnancy disease-specific (e.g. PTBgene, dbPTB) databases. By facilitating the synthesis of diverse functional and evolutionary data in pregnancy-associated tissues and phenotypes and enabling their quick, intuitive, accurate and customized meta-analysis, GEneSTATION provides a novel platform for comprehensive investigation of the function and evolution of mammalian pregnancy.

## INTRODUCTION

Placental mammals, which originated 160 million years ago, uniformly share a conserved set of reproductive traits related to embryonic development within a uterus and nutrient provisioning through a chorioallantoic placenta (1). Paradoxically, this conservation of reproductive mode and function during mammalian evolution is starkly juxtaposed with the evolution of the placenta, one of the most variable of all mammalian organs (2,3). At present, there is no comprehensive explanation for the diversity of evolutionary tempos and modes exhibited by the processes associated with mammalian gestation and pregnancy. The consequences are important not only for our understanding of mammalian pregnancy (4), but also for major features of human evolution, such as the encephalization and bipedalism (5), and how natural selection has acted on and shaped human biology (6,7). And clinically, complications of pregnancy in humans are a major cause of infant mortality around the world (8); for example, complications stemming from birth before term (pre-term birth or PTB), defined in humans as birth before 37 completed weeks of gestation (9), are the leading cause of death in newborns and in children under the age of five (10,11).

Several funding agencies have recognized both the seriousness of pregnancy associated medical problems and the persistence of many unanswered questions about the process. Consequently, they are currently increasing their investments in the study of the biology and pathologies of pregnancy, which will lead to the generation of large amounts of diverse types of data in the next few years. Two notable examples are the NIH-sponsored Human Placenta Project (12), aimed to ‘understand the role of the placenta in health and disease’, and the March of Dimes-sponsored Prematurity Research Centers (http://prematurityresearch.org/), ‘dedicated to solving the mysteries of premature birth’. Because PTB has a significant genetic component (13,14), there is general consensus that emerging molecular and genomic resources provide new opportunities to not only make fundamental advances in our understanding of the evolution and function of mammalian pregnancy (4,15–20), but to also make breakthroughs in treating its diseases (8,21–24).

At present, however, such advances are limited by the fact that such data and resources are dispersed either in many different journals’ supplements or across several different databases, making synthesis of available information slow and costly, and hampering powerful system approaches that involve overlaying diverse data types and analyses in the treatment of disease (25,26). To facilitate this synthesis, we have developed GEneSTATION (http://genestation.org), a database that integrates diverse types of *-omics* data across mammals to advance understanding of the genetic basis of pregnancy-associated phenotypes and to accelerate the translation of discoveries from model organisms to humans. The database's name, GEneSTATION, is a compound word created by blending together ‘gene’ and ‘gestation’; it can be read as ‘gestation’ if the reader considers only the capitalized letters, or as ‘gene station’ if the reader considers all letters, and is intended to highlight the fact that this database is focused on synthesizing information on genes related to gestation.

GEneSTATION provides the data and tools to easily explore pregnancy from three complementary perspectives, evolutionary, organismal, and molecular, at three levels of synthesis. At the first level, individual gene pages integrate the evolutionary, organismal, and molecular perspectives in three easily accessible tabs, providing a comprehensive picture of the breadth of the data available for a single gene and introducing researchers to analyses and data that they have not previously considered. At the second level, individual analysis pages provide access to genome-wide information from a single perspective, such as natural selection in the human lineage, differential expression in complications of pregnancy, or protein-protein interactions among genes known to be involved in pregnancy. At the final level of synthesis, the Gene Set Analysis tool and the novel ‘SynTHy’ (Synthesis and Testing of Hypotheses) tool enable researchers to synthesize on-the-fly the many types of information available through the development and evaluation of testable hypotheses.

## DATA SOURCES AND DATA ORGANIZATION

### Organism life history data

GEneSTATION contains information on pregnancy- and reproduction-associated characteristics for every mammal genome present in the database: human (*Homo sapiens*), elephant (*Loxodonta africana*), chimpanzee (*Pan troglodyte*s), cow (*Bos taurus*), macaque (*Macaca fascicularis*), cat (*Felis catus*), dog (*Canis lupus*), goat (*Capra hircus*), guinea pig (*Cavia porcellus*), horse (*Equus caballus*), mouse (*Mus musculus*), gibbon (*Nomascus leucogenys*), rat (*Rattus norvegicus*), baboon (*Papio anubis*), vole (*Microtus ochrogaster*), rabbit (*Oryctolagus cuniculus*), rhesus monkey (*Macaca mulatta*), sheep (*Ovis aries*), orangutan (*Pongo abelii*), gorilla (*Gorilla gorilla*), marmoset (*Callithrix jacchus*), wild boar (*Sus scrofa*), and platypus (*Ornithorhynchus anatinus*). Specifically, life history characteristics including mean gestation length, neonate development, placental structure and shape, litter size, interbirth interval, adult body mass, maximum longevity and the timing of neonatal brain growth, an interesting characteristic relevant to potential complications of early parturition (27), are provided for each species (for data sources see Materials and Methods).

### Gene-specific data

**Figure 1.**
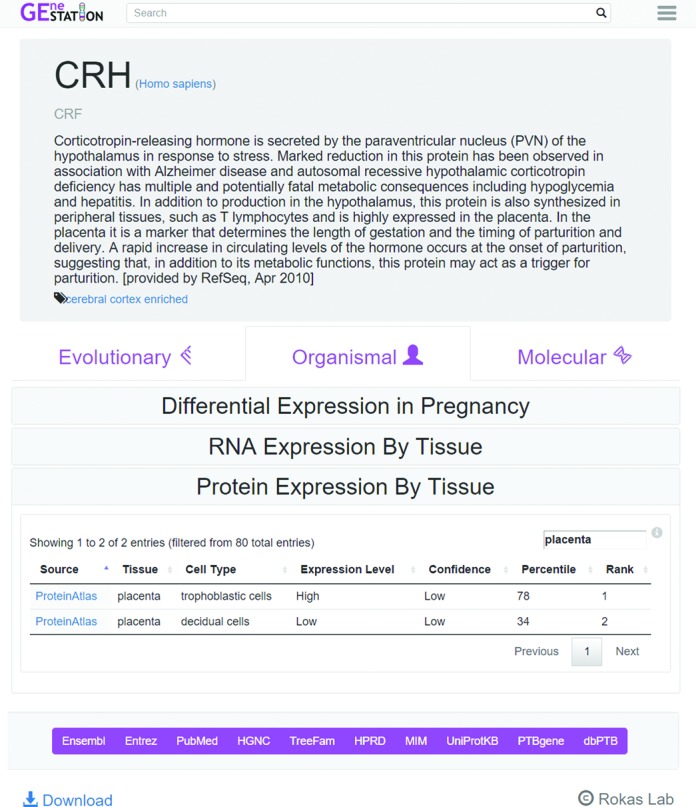
Screen shot of a typical GEneSTATION gene page. Each gene page includes a summary from RefSeq and data organized into three tabs, EVOLUTIONARY, ORGANISMAL and MOLECULAR. In this figure, the ORGANISMAL tab for the CRH gene is open and the Protein Expression by Tissue section is expanded. In this example, the table is filtered by the search term ‘placenta’ and displays the expression levels of CRH protein by the two cell types annotated in this tissue. Links at the bottom of the page provide easy access to other relevant databases. The search bar, which provides instant search, is prominently visible on all pages. Additional analyses, information about GEneSTATION, forms to upload data, and frequently asked questions are accessible by clicking the triple line button on the top right.

In addition to the life history data for the 23 mammals, every gene in each mammalian genome has a page on GEneSTATION that depicts the available evolutionary, organismal and molecular knowledge for that gene, with data from each category reported in a separate tab (Figure 1). The juxtaposition of diverse data is designed to guide users toward a more comprehensive understanding of genes of interest and facilitate serendipitous construction of novel hypotheses. For example, a GEneSTATION user may look up a gene of interest with enriched expression in the placenta and quickly discover that this gene additionally: (i) is often differentially expressed in studies on preeclampsia, a complication of pregnancy characterized by high blood pressure (both of these data types are reported in the organismal tab), (ii) originated coincidentally with the placental mammals (reported in the evolutionary tab) and (iii) interacts with known pregnancy related genes (reported in the molecular tab). Collectively, these associations would suggest that the gene would be a good candidate for further exploration.

The evolutionary category contains a variety of population and evolutionary data on human genes, and in some instances (e.g. ancient selection, orthology) on genes from diverse mammals (see Materials and Methods). These include the strength of recent selection (measured by *F*_ST_) and ancient selection (measured by d*N*/d*S*), a gene's estimated date and lineage of origin, the SNPs from every human gene, and mammalian orthology relationships.

The data in the organismal category include the Online Mendelian Information in Man (OMIM) phenotypes for each human gene, if available, RNA and protein expression across many tissues from Protein Atlas, including several pregnancy related tissues, as well as all differentially expressed genes from 106 genome-wide comparisons from pregnancy studies across gestational tissues (including placenta, cervix, myometrium, decidua, chorion, amnion) and pathologies (including preeclampsia, intrauterine growth restriction, chorioamnionitis and spontaneous preterm birth) (see Materials and Methods).

The data in the molecular category include the gene annotation (28) information for human and seven other mammalian genomes, and the protein interactions, through STRING (29), for proteins from humans and 15 other species.

In addition to the data sets in the three categories listed above, GEneSTATION displays RefSeq summaries for genes as well as gene-specific links to a wide variety of general databases, e.g. Entrez Gene, PubMed, UniProt, TreeFam (30–33), where available. In addition, GEneSTATION contains links to two pregnancy disease-specific databases, dbPTB (http://ptbdb.cs.brown.edu/dbPTBv1.php) and PTBgene (http://ric.einstein.yu.edu/ptbgene/). Both databases are focused on human PTB; dbPTB contains the output from a computational mining of the literature as well as of the KEGG and dbSNP databases to identify studies, pathways and variants associated with candidate disease-risk genes (34), whereas PTBgene represents the summary of the fewer than 100 genes that show genetic association with preterm birth (35).

## DATA PRESENTATION

A number of general purpose or species-centric databases, e.g., Genecards, SGD, WormBase and FlyBASE (36–39), provide access to diverse data sets on individual genes. By design, such databases aim to present the breadth of all available data for a given gene (e.g. http://www.genecards.org/cgi-bin/carddisp.pl?gene=BRCA1), which means that pages of genes that have been extensively studied can become either cumbersome to navigate or saturated with large amounts of different types of data, potentially obscuring the biological interpretation of relationships among the various resources proffered. For ease of access, each major category on the gene page, e.g. evolutionary, is presented on a separate tab that is divided into subsections, e.g. Evolution in Mammals. These subsections are intended to expand and include additional related types of data in future versions of GEneSTATION.

**Figure 2.**
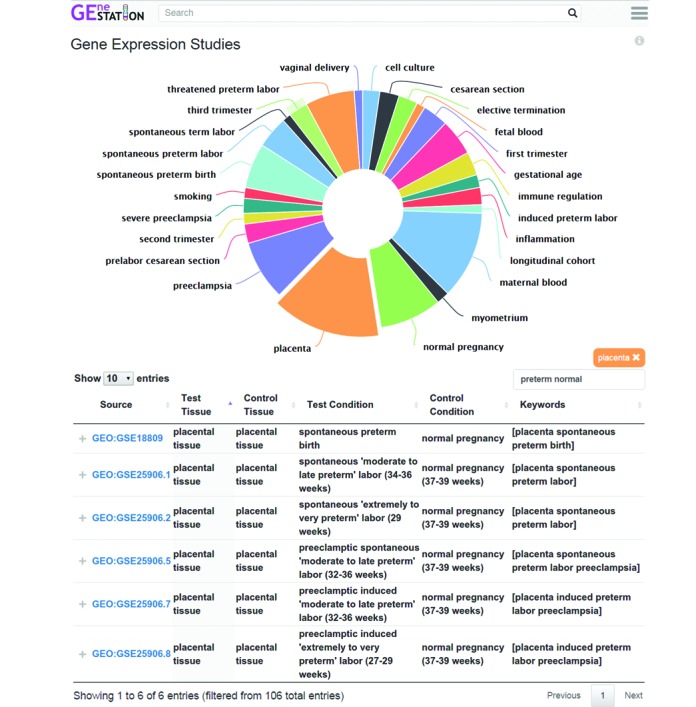
Screen shot of the Gene Expression Studies page. Each analysis page provides a summary figure to help users understand the scope of the data. The Gene Expression Studies page displays the most frequent keywords associated with available studies. The size of each segment in the pie chart is proportional to the number of studies with the keyword. In this case, the user has clicked on the placenta segment of the pie chart, which automatically filters the table for studies involving the placenta. In addition, the user has typed ‘preterm’ and ‘normal’ into the search box, which filters studies based on whether their experimental and control condition descriptions contain these keywords. Each entry in the table provides a link to a page with additional details of the study, the tissue or cell line of the experiment, the test and control conditions, and relevant keywords. The page with study details also includes all genes reported in the study along with expression fold-change and significance.

**Figure 3.**
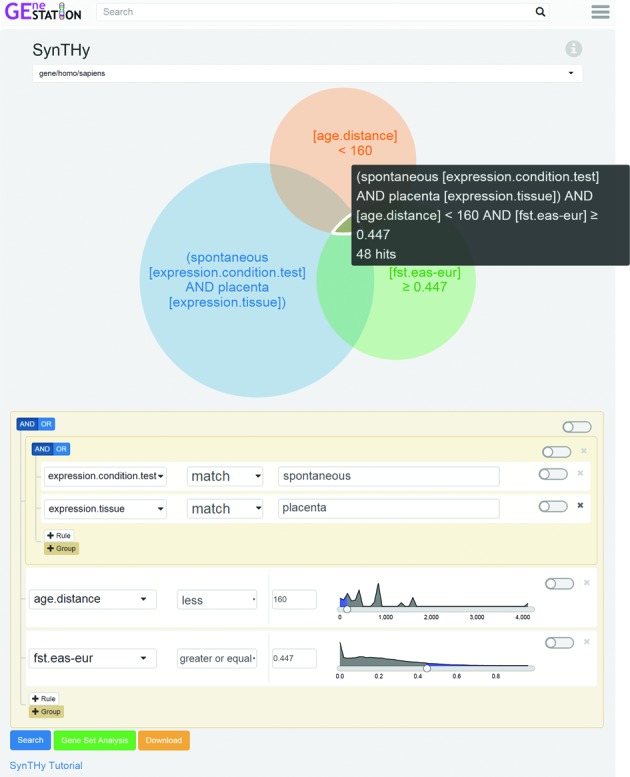
Screen shot of the results of a complex query using the SynTHy tool. The SynTHy tool allows users to form and evaluate hypotheses rapidly, with instant visual feedback guiding exploration. In this example, the user has created a rule group to include genes with significant gene expression differences in studies matching ‘spontaneous’ where the tissue is ‘placenta’. This rule group is visually represented by the blue circle in the Venn diagram. The user has also added a rule to include only genes that arose <160 million years ago (MYA), which is represented in the diagram by a green circle. The distribution of ages is presented to allow users to estimate how many genes are being filtered out. A slider is available to quickly select a cutoff. The final rule selects for genes near SNPs that have very strong differentiation between East Asian populations and European populations (*F*_ST_ ≥ 0.4), which is represented by the orange circle. The distribution to the right of the rule provides users with an estimate of how many genes are being removed by the filter. Users can interact with the Venn diagram to see the number of genes (47 in this example) in each segment along with the search query to find those genes (the black text box). Selecting segments in the Venn diagram, which are scaled to approximate the number of genes, or clicking the search button (in blue, bottom left) will take users to a search page where the genes matching the criteria are listed. The Gene Set Analysis button (in green, bottom left) submits the list of genes matching the intersection of all the rules to the Gene Set Analysis tool to find enrichment for any data stored in GEneSTATION. The Download button (in orange, bottom left) provides users with the selected list of genes and their associated data in JSON format. A screencast tutorial for SynTHy is available in the FAQ page and can be reached from the SynTHy Tutorial link below the search button and from the information icon.

Aided by its focus on a specific biological process, GEneSTATION was developed using state-of-the-art web frameworks to provide a clean visual layout that is easy to interpret and efficient to navigate efficiently (Figures 1–3). For faster access to the data, GEneSTATION is designed to be highly responsive to users and focuses on providing low latency interaction and feedback. We have implemented multiple custom-made visualizations to allow users to quickly grasp the various types of available data and analyses, both for individual genes and across the genome, such as creating interactive summary figures that chart the distributions of the underlying data or studies. For example, the gene expression page (Figure 2) shows the number of studies available by keyword (e.g. ‘myometrium’ or ‘spontaneous preterm birth’), providing instantaneous and meaningful filters of the data, while simultaneously highlighting deficiencies in the number of publically available data sets and identifying opportunities for meta-analyses, as recently described by Eidem *et al*. (40).

Custom-made visualizations are a key design feature of GEneSTATION pages. Examples include a density plot of each analysis in the SynTHy tool (Figure 3), which allows users to quickly select an appropriate cutoff based on the distribution of the values (http://www.genestation.org/SynTHy); the distribution of gene ages plot (http://www.genestation.org/analysis/gene/age), and the distribution of available pregnancy related expression studies by keyword plot (http://www.genestation.org/analysis/gene/expression). To support these custom visualizations, additional html/css/js libraries were included (see Materials and Methods).

## SYNTHESIS AND ANALYSIS

The promise of GEneSTATION is that the rapid exploration of its diverse data types will allow users to generate a synthetic view of the genetic networks underlying pregnancy and its pathologies. Users with lists of genes obtained from experimental results, e.g. differential expression using RNA-seq, but short of fully developed hypotheses, can submit lists of candidate genes for enrichment analysis (http://www.genestation.org/analysis/gene/set) across the various data types (e.g. gene age, tissue expression, differential expression and methylation in disease, GO annotation, protein interactions) and examine statistically significant associations (see Materials and Methods). It has long been recognized that such interactive data exploration phases such as GEneSTATION provides are not only important in the analysis of complex data sets, but also in the formation of new hypotheses (41).

Alternatively, users may visit GEneSTATION with a specific hypothesis about genes involved in a particular process or pathology, or develop one while browsing through the gene pages. With GEneSTATION, finding candidate genes that test a hypothesis has been rendered intuitive and quick by the development of SynTHy (after Synthesize and Test Hypotheses; http://www.genestation.org/SynTHy), a novel tool that goes far beyond typical search tools by visualizing the distribution of the underlying data, giving immediate visual feedback and showing how the various components of a hypothesis impact the resulting list of genes. Rapid exploration of multiple variations on a hypothesis facilitates the development of an integrated view of the genetic relationships underlying the many different data types. For example, a user could ask whether genes with SNPs that have very different frequencies (high *F*_ST_) in populations with high preterm birth rates versus populations with lower preterm birth rates (42) are also preferentially expressed in the placenta or arose in the mammalian ancestor. Any gene list results generated using SynTHy can be easily transferred to the gene set analysis tool for further refinement and exploration. SynTHy thus allows users to ‘find the question’ as readily as to find the answers (41). SynTHy is not intended to fully replace careful statistical analysis using original data but rather to synthesize disparate but high-quality data and analyses and facilitate rapid exploration.

## DATA ACCESS

To facilitate specifically tailored statistical analyses, GEneSTATION makes all data available for easy download in JSON format using the download button on the bottom left of each gene page, analysis page and SynTHy result page.

## SUMMARY AND FUTURE PERSPECTIVES

Understanding the complex functional landscape of pregnancy, how abnormalities of pregnancy arise, or how the biological mechanisms of gestation evolve and translate between species can be greatly augmented by the integration and synthesis of multiple types of experimental data, genomic data, and evolutionary analyses. Importantly, such genome-scale data sets are becoming more frequent and current funding priorities will only accelerate this trend. Consequently, populating GEneSTATION with additional high-quality evolutionary, organismal and molecular data sets is an active and ongoing process, with transcriptomic, proteomic, and imaging data being a high priority. In parallel, we are developing algorithms that will point users interested in a particular gene to other genes or biological processes with similar functional or evolutionary characteristics. Furthermore, GEneSTATION's integration of evolutionary and experimental data will support the development of algorithms that evaluate the likelihood that those specific biological systems or medical interventions that work in a model organism such as mouse or macaque will also work in human pregnancy.

In summary, GEneSTATION facilitates integrative analyses that draw from many types of data, providing a novel platform and paradigm for comprehensive understanding of pregnancy across mammals. It is our hope that GEneSTATION's synthesis becomes a catalyst for the identification and evaluation of candidate genes by biologists interested in the function and evolution of mammalian pregnancy, as well as its complications. More generally, GEneSTATION's synthetic focus on a specific biological process has the potential to become a model for databases aimed at synthesizing the diverse types of biology's ‘big data’ for a wide variety of biological processes.

## MATERIALS AND METHODS

### Publically available data on GEneSTATION

Publically available data that did not need reanalysis or normalization (e.g. gene age, dN/dS, Protein Atlas) were added to GEneSTATION without modification. Details about these data are listed in the online methods page along with links to the original data source. In addition, the methods page describes in more detail data sets that may be difficult to interpret for non-specialists or that has potential caveats for interpretation. Small information icons on each analysis page or in the relevant subsection of gene pages provide links to both the methods page and the original data.

### Sources of organism life history data

Mean gestation length and standard deviations were taken from primary accounts in the research literature focused on reproductive characteristics of each individual species. Neonate development state was either recorded directly from the literature or inferred using average litter size as a proxy (43). For all species, placental type and shape were taken from (1). For non-primates, adult body mass was taken from the PanTHERIA database, as well as average litter sizes, interbirth intervals and maximum longevity for many species (44). For primates, body mass data specifically reflect adult female body mass (45). Finally, data on the timing of brain growth across mammals were taken from (46).

### Variant reanalysis

To provide consistent genome wide analyses of human genetic variation, variant call format (VCF) files, with coordinates lifted to genome build GRCh38, were downloaded from the 1000 Genomes Project FTP site (ftp://ftp-trace.ncbi.nih.gov/1000genomes/ftp/), representing variants for all 2504 unrelated individuals in the 1000 Genome Project Phase 3 cohort (47). Variants were filtered to exclude non-SNP variants, fixed sites, and sites with uncalled or unphased genotypes. Variants were validated against dbSNP build 144 (48) using the ValidateVariants tool in Genome Analysis Toolkit (49). VCFtools (50) was used to calculate pairwise *F*_ST_ statistics (51).

### Microarray reanalysis

We reanalyzed all microarray datasets that were downloaded from NCBI's Gene Expression Omnibus (GEO) (52) using the R package GEOquery. Ambiguous probes that map to multiple genes were discarded. For multiple probes mapping to a single gene, the probe with median significance value was reported. Pairwise differential expression statistics were computed using the eBayes algorithm in the *limma* package.

### Database design

The data for GEneSTATION is stored in PostgreSQL, a highly reliable and durable open source database. For consistent integration of multiple types of data, we use a highly normalized and non-redundant biology focused schema, Chado (53), which is collaboratively developed by the Generic Model Organism Project (54). We have written extensions to this schema so that GEneSTATION can handle the large numbers of genomes and diverse associated data (currently 3.4Tb) already loaded as well as those to be added in the future.

GEneSTATION's large datasets have required the development of high-performance custom tools for data loading, which are built using C++ with libpq, and allow loading of genome-wide datasets in seconds and SNP datasets (e.g. 1000 genomes) in minutes. Python with SQLAlchemy provides a flexible pipeline for loading the highly varied data sources in GEneSTATION, annotations, database cross-references, and controlled vocabularies. GEneSTATION loads both standard data formats, e.g. GFF, FSTA, OBO, and generic formats using JSON files for metadata and tab-delimited files for the data, which facilitates integration of diverse analysis pipelines.

GEneSTATION also uses Elasticsearch, a distributed, in-memory search engine for full-text search and as a store for precalculated, denormalized SQL queries that are computationally intensive. Elasticsearch allows GEneSTATION to respond to advanced queries from users, including boolean and full-text, with lower latency and higher throughput than is possible with PostgreSQL alone.

### Web interface

The web interface to the GEneSTATION database is delivered by a high performance custom server written in Go, a new language developed at Google to power their web services. The server handles querying both PostgreSQL (using sqlx) and Elasticsearch (using elastic), parsing user input, and performing custom analyses on-the-fly (e.g., analysis of user submitted gene sets). The server provides an NCBI-like query language to query the Elasticsearch search engine and provides uniform access to all GEneSTATION data via a REST interface. The server caches most pages, reducing latency to much less than a second in most cases.

The foundation of the user interface is Bootstrap, an integrated library of html elements, css, and javascript, which allows consistent visual layouts even on mobile devices and provides tools for rich user interaction, e.g. tabs, tooltips, popovers, while supporting users on both handheld devices (e.g. iPads and iPhones) and older browsers. Additional functionality was achieved with specialized html/css/js libraries, which include: autocompeter.js for immediate search results feedback and autocomplete; multiple libraries, selectize.js, autosize.js, d3.js, venn.js, and react.js where used to build the SynThy tool; multiple libraries, jquery.js, jquery.form.js, responsive-bootstrap-toolkit.js, jquery-highlighttextarea.js, jquery-hoverIntent.js and rPage.js, were used for simpler JavaScript development and richer user interaction.

### Visualization

Data in charts and graphs are presented using highcharts.js, data in table formats are displayed with jquery.dataTables.js and the interactive organisms page uses jquery.mixitup.js.

### Gene set analysis

*P*-values for gene set analyses are calculated using the cumulative hypergeometric distribution (similar to Fisher's Exact Test). The background is adjusted for each set to match the number of genes reported in each data set, either analysis or annotation.
